# Supplementary material for: Assessment of a multiplex arbovirus PCR Detection Test in an area endemic for Chikungunya, Zika, and Dengue viruses: An evaluation of kit performance characteristics in line with Clinical Laboratory Improvement Amendments (CLIA) Standards
Source: PLoS One. 2025 Jun 24;20(6):e0309626. doi: 10.1371/journal.pone.0309626 (PMC12186890; doi:10.1371/journal.pone.0309626)
Supplement: Attachment — Submitted filename: Response to reviewer.doc [file pone.0309626.s002.doc]

[Leonard Kingwara]

[Kenya National Public Health Institute]

[20750-00202]

[19^th^ Dec 2024]

Dear Editor,

Re: Performance Evaluation of a Multiplex PCR Assay for Chikungunya, Zika, and Dengue Detection: CLIA Standards Assessment in a Partially Endemic Region

Dear Editor,

Sincerest thanks for your response and reviewers’ comments on our manuscript. We sincerely apologize for the great time it has taken us to respond to these comments and hope that a revised version of the manuscript will still be considered by *PLOS one*. We have modified the paper in response to the extensive and insightful reviewer comments. In the abstract section we have added additional results to give the manuscript proper angulation and clarity to fully address the reviewer’s comments. In addition, we have rewritten sections of the manuscript and revised the title. We will thus not respond point by point as nearly the entire manuscript has been overhauled.

Thank you for your consideration of this manuscript.

Sincerely,

Leonard Kingwara
